# Beyond HbA1c: An Analytical Cross-Sectional Study of the Prevalence and Determinants of Poor Glycemic Control Among Adults With Type 2 Diabetes Mellitus in Puducherry, India

**DOI:** 10.7759/cureus.115488

**Published:** 2026-08-31

**Authors:** Roselin Mohandas, Rajesh K Konduru, Velavan Anandan, Prahankumar R, Manikandan Mani, Anil J Purty

**Affiliations:** 1 Community Medicine, Pondicherry Institute of Medical Sciences, Puducherry, IND; 2 Community Medicine, Mahatma Gandhi Medical College and Research Institute, Puducherry, IND

**Keywords:** determinants, hba1c, prevalence, treatment adherence, type 2 diabetes mellitus

## Abstract

Background: Diabetes mellitus is characterized by chronic hyperglycemia resulting from defects in insulin secretion, insulin action, or both. It is one of the major causes of premature deaths, which occurs on account of its complications due to uncontrolled diabetes. This demands an urgent need for strategies to control the emerging epidemic of diabetes. The objectives of this study were to estimate the proportion of poor glycemic control and to identify the factors influencing poor glycemic control among adults with type 2 diabetes mellitus in Puducherry district.

Methods: A community-based cross-sectional analytical study was conducted among 340 individuals with type 2 diabetes mellitus who had the disease for at least six months of duration in Puducherry district. A list of individuals who underwent HbA1c estimation at the biochemistry laboratory of a tertiary care hospital was obtained prospectively. The eligible participants were contacted and visited at their residences. A semi-structured questionnaire was used to collect sociodemographic, behavioral, and clinical profiles.

Results: The proportion of people with poor glycemic control was 217 (63.8%). Determinants such as age, place of residence, alcohol consumption, and treatment adherence were found to be associated with poor glycemic control. However, when adjusted for confounders, irregular treatment adherence emerged as the only independent factor associated with poor glycemic control (adjusted odds ratio (AOR): 19.54; confidence interval (CI): 7.65-49.90, P-value = 0.000).

Conclusions: This study emphasizes the importance of treatment adherence at the primary care level in order to substantially improve glycemic outcomes and reduce diabetes-related complications.

## Introduction

Diabetes mellitus (DM) is a chronic metabolic disorder characterized by persistent hyperglycemia and resulting from defects in insulin secretion, insulin action, or both [1]. Type 2 DM (T2DM) accounts for approximately 90% of all diabetes cases and has emerged as a major global public health concern, driven by rapid urbanization, lifestyle transitions, and population aging [2,3].

According to International Diabetes Federation (IDF) Diabetes Atlas (2025) reports, approximately 590 million adults (11.1%) worldwide are living with diabetes; this number is projected to reach 853 million by 2050 [4]. India bears a substantial share of this burden, with an estimated prevalence of ~8.9 crores (10.5%) among adults [5] and projections indicating a steep rise in the upcoming decades [3].

National surveys such as the National Family Health Survey (NFHS)-5 (2019-2021) report elevated blood sugar levels among 97,800 (13.5%) women and 15,900 (15.6%) men in India [6], with higher prevalence observed in southern states including Tamil Nadu and Puducherry [7]. The Indian Council of Medical Research-India Diabetes (ICMR-INDIAB) study further highlights a disproportionately high burden of diabetes and prediabetes in these regions [8].

Glycated hemoglobin (HbA1c) is a reliable indicator of long-term glycemic control, reflecting average blood glucose levels over the preceding 2-3 months [9]. Evidence from landmark studies has demonstrated that each 1% reduction in HbA1c significantly reduces the risk of diabetes-related complications [10].

Glycemic control in individuals with T2DM is influenced by multiple factors, including sociodemographic characteristics, disease duration, lifestyle behaviors, and treatment adherence. Despite high prevalence, community-level evidence on determinants of glycemic control in Puducherry is limited. Therefore, this study was undertaken to estimate the proportion of poor glycemic control and identify factors influencing glycemic control among adults with T2DM in Puducherry, with the aim of preventing diabetes-related complications and fostering a healthier cum resilient community.

## Materials and methods

A community-based cross-sectional analytical study was carried out among adults with T2DM residing in rural and urban areas of Puducherry district. Data collection was carried out from May 2019 to September 2020. Individuals aged ≥21 years with a confirmed diagnosis of T2DM for at least six months and residing in Puducherry were included. Bedridden individuals and those unavailable after two consecutive household visits were excluded. The sample size was calculated using the formula $n = 4pq/d^{2}$, assuming a prevalence of poor glycemic control of 63% based on a previous study, with a 95% confidence level and 9% relative precision [11]. After adjusting for a 15% nonresponse rate, the required sample size was found to be 328, which was rounded off to 340.

A line list of individuals undergoing HbA1c estimation by high-pressure liquid chromatography using the Variant II Turbo machine (BIORAD, Hercules, CA, USA) method was obtained prospectively since May 2019 at the biochemistry laboratory (National Accreditation Board for Testing and Calibration Laboratories (NABL)-accredited) of a tertiary care hospital. The eligible participants were contacted and visited at their residences. Recruitment was continued until the required sample size was achieved. The objectives were to estimate the proportion of poor glycemic control (HbA1c ≥ 8%) and to identify the factors influencing poor glycemic control among adult individuals (aged 21 years and above) with T2DM residing in Puducherry district.

Data were collected using a pretested semi-structured questionnaire (Appendix). Information obtained on sociodemographic characteristics, clinical characteristics, behavioral risk factors (smoking, alcohol use, dietary practices, and physical activity), and treatment adherence were self-reported by the study participants. Physical activity was assessed using the WHO STEPS questionnaire and was categorized into low, moderate, and high [12]. Waist circumference was measured using standardized procedures, and the HbA1c values were obtained from laboratory records. The dependent variable was poor glycemic control. Independent variables included age, gender, educational status, occupation, residence, family history of diabetes, behavioral risk factors, duration of diabetes, and treatment adherence.

Operational definitions

Poor Glycemic Control

Glycemic control was categorized as poor glycemic control (HbA1c ≥ 8%) and good glycemic control (HbA1c < 8%) based on national guidelines [3].

Adherence to Medication

Adherence (compliance) is the degree to which a patient follows a treatment regimen. For medications, adherence requires that the medication be taken as prescribed in terms of dose, dosing interval, duration of treatment, and any additional special instructions with respect to the time of the meal [13].

Physical activity categories

Vigorous Activities

Vigorous activities are defined as work that involves vigorous activity like lifting heavy loads, digging, and construction [12].

Moderate Activities

Moderate activities are defined as work that involves moderately intense activity that causes a small increase in breathing or heart rate (such as brisk walking or carrying light loads, recreation, or exercise) for at least 10 minutes [12].

Mild Activities

Mild activities were defined as walking or bicycling for at least 10 minutes to travel from place to place, as well as activities involving recreation or exercise [12].

Data were entered in Microsoft Excel (Microsoft Corp., Redmond, WA, USA), and analysis was performed using STATA version 17 (StataCorp LLC, College Station, TX, USA). Continuous variables were expressed as mean ± standard deviation (SD), and categorical variables as frequencies and percentages. The association between independent variables and glycemic control was assessed using the Chi-squared test. Variables with P-value ≤ 0.1 in univariate analysis were included in multivariate logistic regression to identify independent determinants. Adjusted odds ratios (AORs) with 95% confidence intervals (CIs) were calculated. P-value < 0.05 was considered statistically significant.

The study was approved by the Research Committee, and ethical clearance was obtained from the Institute Ethics Committee (IEC number: RC/18/67 dated November 2, 2018). Written informed consent was obtained from all participants prior to data collection.

## Results

A total of 340 adults with T2DM were included in the study. The mean (±SD) age of the participants was 56.5 ± 10.9 years, and the majority of them were men (201 (59.1%)). The sociodemographic characteristics of the study participants are presented in Table 1.

**Table 1:** Sociodemographic characteristics of study participants (n = 340).

Baseline characteristics	Frequency (%)
Age (in years)	
31-45	62 (18.2)
46-60	165 (48.6)
>60	113 (33.2)
Gender	
Male	201 (59.1)
Female	139 (40.9)
Marital status	
Married	322 (94.7)
Single	18 (5.3)
Educational status	
Illiterate	54 (15.9)
Middle school	123 (36.2)
High school	98 (28.8)
Graduate	65 (19.1)
Occupation	
Professional	28 (8.2)
Semi-professional	132 (38.8)
Arithmetic skill jobs	61 (18.0)
Skilled worker	119 (35.0)
Place of residence	
Urban	187 (55.0)
Rural	153 (45.0)

The clinical characteristics of the participants are shown in Table 2. One-third of the participants had diabetes for more than 10 years. Oral hypoglycemic agents (OHAs) were the most commonly prescribed treatment modality, followed by combined insulin and OHA therapy. Overall, two-thirds of the participants reported irregular treatment adherence.

**Table 2:** Clinical characteristics of study participants (n = 340).

Clinical characteristics	Frequency (%)
Duration of diabetes	
6 months to 1 year	33 (9.7)
1 year to 3 years	55 (16.2)
3 years to 5 years	70 (20.6)
5 years to 10 years	69 (20.3)
>10 years	113 (33.2)
Type of treatment	
Only lifestyle modification	3 (0.9)
Insulin	27 (7.9)
Oral hypoglycemic agents (OHAs)	265 (77.9)
Insulin + OHAs	39 (11.5)
AYUSH	1 (0.3)
Others	5 (1.5)
Treatment compliance	
Regular	106 (31.2)
Irregular	234 (68.8)

The mean (±SD) HbA1c level of the study participants was 9.42% ± 2.83%. Based on the predefined HbA1c cut-off of 8%, 217 (63.8%) participants had poor glycemic control, whereas 123 (36.2%) had good glycemic control. Behavioral and lifestyle characteristics are summarized in Table 3.

**Table 3:** Risk factor distribution among study participants (n = 340).

Risk factors	Frequency (%)
History of diabetes in the family	
Yes	172 (50.6)
No	168 (49.4)
Tobacco smoking	
Yes	32 (9.4)
No	308 (90.6)
Alcohol intake	
Yes	60 (17.6)
No	280 (82.4)
Dietary modification	
Yes	307 (90.3)
No	33 (9.7)
Physical activity	
Yes	250 (73.5)
No	90 (26.5)

Univariate logistic regression analysis identified age, marital status, place of residence, alcohol consumption, and treatment adherence as variables showing an association with glycemic control (Table 4). However, only treatment adherence was significantly associated with poor glycemic control. Participants with irregular treatment adherence had more than 20-fold higher odds of poor glycemic control compared with those who reported regular adherence (crude OR (COR): 20.54; CI: 8.07-52.27; P = 0.000).

**Table 4:** Univariate analysis of factors influencing glycemic control. *As per WHO, recommended waist circumference cut-offs for Asian population (men ≥ 90 cm, women ≥ 80 cm). OR: odds ratio; CI: confidence interval

Variables	Categories	Frequency (%)		Crude OR (CI)	P-value
		Poor control	Good control		
Age	31-45	46 (74.2)	16 (25.8)	1	
	46-60	103 (62.4)	62 (37.6)	0.57 (0.30-1.10)	0.098
	>60	68 (60.2)	45 (39.8)	0.52 (0.26-1.03)	0.065
Gender	Male	129 (64.2)	72 (35.8)	1	
	Female	88 (63.3)	51 (36.7)	0.96 (0.61-1.50)	0.870
Marital status	Married	209 (64.9)	113 (35.1)	1	
	Single	8 (44.4)	10 (55.6)	0.43 (0.16-1.12)	0.086
Educational status	Illiterate	36 (66.7)	18 (33.3)	1	
	Middle school	83 (67.5)	40 (32.5)	1.03 (0.52-2.04)	0.915
	High school	60 (61.22)	38 (38.8)	0.78 (0.39-1.58)	0.506
	Graduate	38 (58.5)	27 (41.5)	0.70 (0.33-1.49)	0.359
Occupation	Professional	15 (53.6)	13 (46.4)	1	
	Skilled	91 (68.9)	41 (31.1)	1.92 (0.83-4.40)	0.122
	Unskilled	40 (65.6)	21 (34.4)	1.65 (0.66-4.10)	0.281
	Unemployed	71 (59.7)	48 (40.3)	1.28 (0.56-2.93)	0.557
Residence	Urban	112 (59.9)	75 (40.1)	1	
	Rural	105 (68.6)	48 (31.4)	1.46 (0.93-2.29)	0.096
Family h/o diabetes	Yes	112 (65.1)	60 (34.9)	1.12 (0.71-1.74)	0.616
	No	105 (62.5)	63 (37.5)	1	
Tobacco smoking	Yes	21 (65.6)	11 (34.4)	1.09 (0.50-2.34)	0.824
	No	196 (63.6)	112 (36.4)	1	
Alcohol consumption	Yes	43 (19.8)	17 (13.8)	1.54 (0.83-2.83)	0.166
	No	174 (80.2)	106 (86.2)	1	
Physical activity	No	71 (65.7)	37 (34.3)	1.13 (0.70-1.82)	0.616
	Yes	146 (62.9)	86 (37.1)	1	
Diet modification	Yes	193 (62.9)	114 (37.1)	1	
	No	24 (72.7)	9 (27.3)	1.57 (0.70-3.50)	0.266
Stress management	No	69 (64.5)	38 (35.5)	1.04 (0.64-1.68)	0.863
	Yes	148 (63.5)	85 (36.5)	1	
History of comorbidity	Present	13 (68.4)	6 (31.6)	1.24 (0.46-3.35)	0.668
	Absent	204 (63.5)	117 (36.5)	1	
Duration of diabetes	6 months-1 year	23 (69.7)	10 (30.3)	1	
	1-3 years	32 (58.2)	23 (41.8)	0.60 (0.24-1.51)	0.282
	3-5 years	43 (61.4)	27 (38.6)	0.69 (0.28-1.67)	0.416
	5-0 years	41 (59.4)	28 (40.6)	0.63 (0.26-1.54)	0.317
	>10 years	78 (69.0)	35 (31.0)	0.96 (0.41-2.25)	0.942
Treatment compliance	Regular	101 (95.3)	5 (4.7)	1	
	Irregular	116 (49.6)	118 (50.4)	20.54 (8.07-52.27)	0.000
Waist circumference*	Abnormal	61 (65.6)	32 (34.4)	1.11 (0.67-1.83)	0.677
	Normal	156 (63.2)	91 (36.8)	1	

Variables with P-value ≤ 0.1 in the univariate analysis were entered into a multivariable logistic regression model (Table 5). After adjustment for potential confounding factors, irregular treatment adherence remained the only independent factor associated with poor glycemic control (AOR: 19.54; 95% CI: 7.65-49.90; P = 0.000).

**Table 5:** Multivariable logistic regression analysis to determine the independent factors of glycemic control. OR: odds ratio; CI: confidence interval

Variables	Categories	Adjusted OR (CI)	P-value
Age	31-45	1	
	46-60	0.65 (0.31-1.36)	0.255
	>60	0.71 (0.32-1.56)	0.403
Residence	Urban	1	
	Rural	1.37 (0.82-2.28)	0.221
History of alcohol consumption	Yes	1.25 (0.62-2.54)	0.522
	No	1	
Treatment compliance	Irregular	19.54 (7.65-49.90)	0.000
	Regular	1	

## Discussion

The present study demonstrated that 217 (63.8%) adults with T2DM had poor glycemic control, indicating a substantial burden of uncontrolled diabetes among patients attending a tertiary care teaching hospital in Puducherry. Despite considerable advances in diabetes management, achieving optimal glycemic control remains a major challenge. The observed high prevalence highlights the need for strengthening diabetes management strategies to prevent complications and maintain quality of life.

Studies from Kerala have reported poor glycemic control among 58%-79% of patients with T2DM [14,15], while studies from Jordan and China have reported more than half of the participants with poor glycemic control [16,17], although slightly lower proportions have been described in Ethiopia [18]. The observed variation across studies may be attributed to differences in study settings, participant characteristics, HbA1c thresholds used to define poor control, access to healthcare services, adherence to treatment, and socioeconomic conditions. Nevertheless, the consistently high prevalence across studies underscores the persistent challenge of achieving recommended glycemic targets.

The mean age of the participants in the present study was 56.50 ± 10.90 years, which was comparable with findings from Najeeb et al., Kankonkar et al., and several other studies, reflecting the increasing burden of T2DM among middle-aged and older adults [14,19]. Although participants aged 46 years and above demonstrated lower odds of poor glycemic control in the univariate analysis, the association was not statistically significant after adjustment. Supportive findings were observed in the study done by Eticha et al., in which younger age was identified as a factor associated with poor glycemic control [18], whereas contradictory study findings by Hariyani demonstrated poorer glycemic control among older adults [20]. The inconsistent relationship between age and glycemic control may reflect differences in disease duration, treatment intensity, healthcare utilization, and self-management practices across different populations.

Although women demonstrated slightly lower odds of poor glycemic control, the difference was not statistically significant. The findings were similar to those reported by Al-Qerem et al. and Anupama et al., who found that women had significantly higher odds of having controlled diabetes and that poor glycemic control was common in men [16,21]. This finding contrasts with a study by Najeeb et al. in which female gender was a risk factor for poor glycemic control [14]. These discrepancies may be explained by sociocultural differences, healthcare-seeking behavior, treatment adherence, and lifestyle practices across study populations.

Participants with higher educational attainment tended to have lower odds of poor glycemic control, although the association did not reach statistical significance. The findings were similar to those reported by Hariyani, Tarekegn et al., and Fiseha et al., in which lower educational status was associated with poor glycemic control among participants [20,22,23]. Education is known to influence health literacy, self-care practices, and treatment adherence; however, the absence of statistically significant associations in the present study suggests that demographic characteristics alone may not suffice to find an association with glycemic control; hence, considering behavioral and clinical determinants is essential.

The present study shows that those participants who were single were found to have lower odds of poor glycemic control (COR 0.43, CI = 0.16-1.12), participants residing in rural areas had higher odds of poor glycemic control (COR 1.46, CI = 0.93-2.29), and both approached statistical significance with P-values of 0.086 and 0.096, respectively. However, marital status and place of residence were not independently associated with glycemic control. This could be attributed to the multiple responsibilities of married individuals, which may make it difficult for them to follow measures to maintain good glycemic status, and for those participants residing in rural areas, challenges may be due to differences in healthcare access and economic barriers. This demands more focus on health in rural settings.

In this study, participants who reported alcohol consumption and tobacco use demonstrated higher odds of poor glycemic control, although these associations were not statistically significant after adjustment. A similar study finding was reported by Anupama et al. [21]. A study by Blah et al. reported a contradictory finding that alcohol consumption was not associated with poor control [24]. Alcohol may adversely influence glycemic control through poor medication adherence, unhealthy dietary practices, hepatic metabolic alterations, and reduced compliance with recommended lifestyle measures. Although the association did not achieve statistical significance in our study, the findings suggest that alcohol use remains an important modifiable behavioral risk factor that warrants attention during routine diabetes care.

Participants who did not engage in regular physical activity, dietary modification, and stress management measures had higher odds of poor glycemic control. However, their respective P-values were not statistically significant. The findings were similar to those reported by Hariyani and Anupama et al., which have consistently demonstrated better glycemic outcomes among individuals practicing regular exercise and appropriate dietary modification [20,21]. The lack of statistical significance in the present study may reflect the self-reported nature of lifestyle assessment, variability in the intensity and duration of physical activity, and differences in dietary adherence that were not quantitatively measured.

Family history of diabetes showed a modest positive association with poor glycemic control. However, this relationship was not statistically significant. A similar finding was reported by a study by Anupama et al., which reported statistical significance between family history of diabetes and poor glycemic control [21]. A study by Blah et al. has reported conflicting findings regarding its association with glycemic control [24]. Genetic predisposition alone is unlikely to determine glycemic status without the influence of behavioral and therapeutic factors. Therefore, comprehensive diabetes management should continue to emphasize lifestyle modification irrespective of family history.

Clinical characteristics including duration of diabetes and presence of comorbidity also demonstrated positive associations with poor glycemic control, although none emerged as independent factors after adjustment. Hariyani reported similar findings with respect to the presence of comorbidity [20]. The findings reported by Wang et al., Tarekegn et al., and Phuwilert et al. reported duration of diabetes as a key factor of poor glycemic control [17,22,25]. In the present study, central adiposity showed higher odds of poor glycemic control; however, this relationship was not statistically significant. This finding was similar to the study done by Kankonkar et al. in which increased abdominal girth was linked to higher HbA1c values [19].

Longer duration of diabetes has been consistently associated with progressive β-cell dysfunction, increasing insulin resistance, and declining glycemic control. Similarly, increased waist circumference has been an established marker of insulin resistance and metabolic dysfunction. Although these variables were not statistically significant in the present study, the observed direction of association is biologically plausible and consistent with existing evidence. The absence of statistical significance may be attributable to heterogeneity in treatment patterns, differences in disease severity, or insufficient statistical power to detect relatively small effect sizes.

Among all the variables examined, irregular treatment adherence emerged as the strongest and only independent factor associated with poor glycemic control. Participants with irregular treatment adherence had substantially higher odds of poor glycemic control even after adjustment for potential confounding factors (AOR 19.54, CI = 7.65-49.90, P-value = 0.000). Study findings by Najeeb et al., Phuwilert et al., and Otaif and Bahari reported irregular treatment adherence to be positively associated with poor glycemic control and were found to be statistically significant [14,25,26]. Although the AOR was large, the accompanying wide CI suggests limited precision of the estimate, which may be related to the distribution of participants across adherence categories. Nevertheless, the direction and strength of the association are consistent with existing literature and reinforce the importance of treatment adherence in diabetes management. Medication adherence remains a modifiable determinant that can be effectively addressed through patient education, regular follow-up, simplified treatment regimens, counseling, and family support.

The strengths of the study are that it has evaluated the determinants of glycemic control using HbA1c, an objective indicator of long-term glycemic status. Multivariable logistic regression was employed to identify independent factors while accounting for potential confounding. Inclusion of participants from both rural and urban areas enhances the relevance of the findings to routine clinical practice in similar healthcare settings.

The study has certain limitations. First, facility-based sampling with household follow-up was done, which may limit the generalizability of the findings to the wider community. Second, behavioral variables such as dietary practices, physical activity, and treatment adherence were assessed using self-reported information and are therefore subject to recall and social desirability bias.

## Conclusions

The present study demonstrated that nearly two-thirds of adults with T2DM attending a tertiary care teaching hospital had poor glycemic control. The findings emphasize that several factors may influence glycemic status, such as age, place of residence, alcohol consumption, and treatment compliance. However, when adjusted for confounders, treatment adherence was found to be significantly associated. Hence, in practice, it is recommended that a comprehensive understanding of factors associated with poor glycemic control in a similar setting is important, and interventions targeted toward these risk factors are essential to achieve glycemic control.

## Appendices

Questionnaire

Proforma No.:

Factors influencing glycemic control among adults with type 2 diabetes mellitus in Puducherry district

Sociodemographic details:

1. Name:

2. Age & date of birth:

3. Gender:

4. Habitat:

(1) Urban  (2) Rural

Address:

Mobile:

5. Educational status of diabetic individuals:

(1) Illiterate  (2) Middle School  (3) High School  (4) Graduate

6. Marital status:

(1) Married  (2) Unmarried  (3) Widowed  (4) Divorced

7. Occupation:

(1) Professional  (2) Semi-professional  (3) Arithmetic skill jobs  (4) Skilled worker  (5) Semi-skilled  (6) Unskilled worker  (7) Unemployed  (8) Housewife  (9) Student  (10) Retired

8. Total family income per month:

9. Number of family members:

Clinical characteristics (diabetes-related history):

10. Time of diagnosis: __ month __ year

11. For how many years have you been suffering from diabetes?

(1) 6 months to 1 yr  (2) 1 to 3 yrs  (3) 3-5 yrs  (4) 5-10 yrs  (5) >10 years

12. Type of treatment for diabetes:

(1) Only lifestyle modification  (2) Insulin  (3) OHAs  (4) Insulin + OHAs  (5) AYUSH (specify) ____  (6) Others (specify) ____

13. Duration of treatment:

(1) 6 months-1 yr  (2) 1-3 yrs  (3) 3-5 yrs  (4) 5-10 yrs  (5) >10 years

14. Are you taking OHAs regularly? (1) Regular  (2) Occasionally irregular  (3) Irregular

15. For those on OHAs, which group of drugs are you taking?

(1) Biguanides  (2) Sulfonylureas  (3) Others (specify)  (4) Combination of 1,2 OHAs  (5) Combination of 1,2,3 OHAs

16. Are you on insulin injections?

(1) Yes  (2) No

17. If yes, how long have you been on insulin injections?

(1) 6 months to 1 yr  (2) 1-3 yrs  (3) 3-5 yrs  (4) >5 yrs

18. What type of insulin are you taking?

(1) Short-acting  (2) Intermediate-acting  (3) Combination of both  (4) Premixed  (5) Insulin analogue

Risk factors:

19. Family history of diabetes:

(1) Yes  (2) No

Smoking:

20. Do you currently smoke any tobacco products such as cigarettes, beedi?

(1) Yes  (2) No (If No, go to question no. 24)

21. Do you currently smoke tobacco daily?

(1) Yes  (2) No (If No, go to question no. 24)

22. Years of smoking? __ years

23. On average, what number of the following items do you smoke per day?

Cigarettes ____  Bidis ____  Cigars ____  Others ____

24. In the past, did you ever smoke daily?

(1) Yes  (2) No (If No, go to question no. 26)

25. How long has it been since you stopped smoking daily? __ years __ months

26. Do you currently use any smokeless tobacco products such as snuff, chewing tobacco, or betel?

(1) Yes  (2) No  (If No, go to question no. 29)

27. Do you use smokeless tobacco products daily?

(1) Yes  (2) No (If No, go to question no. 29)

28. On average, how many times a day do you use?

Snuff ____  Chewing tobacco ____  Betel quid ____  Others ____

Alcohol consumption:

29. Have you ever had a drink that contains alcohol?

(1) Yes  (2) No (If No, go to question no. 33)

30. Have you consumed an alcoholic drink within the past 12 months?

(1) Yes  (2) No (If No, go to question no. 33)

31. At what age did you first start drinking regularly (at least once a month)? __ years

1 standard drink =

__ 30 mL spirits (whisky, rum, brandy, gin, vodka)

__ 330 mL (1/2 bottle) beer

__ 110 mL (1/3 bottle) strong beer

__ Glass of handia/toddy

32. How often have you had a drink containing alcohol in the past 12 months?

(1) Daily  (2) 1-3 days a week  (3) 4-6 days a week  (4) 1-3 days per month  (5) Less than once a month

Comorbidity:

33. Do you have a history of any of the following comorbidities?

(1) HTN  (2) CAD  (3) Nephropathy  (4) Retinopathy  (5) Diabetic foot  (6) Dyslipidemia  (7) Obesity  (8) TB  (9) Others (specify) ____

34. Are you taking any medications for comorbidities?

35. If yes, are you taking the medication regularly?

(1) Regular  (2) Occasionally irregular  (3) Irregular

Diet:

36. Are you following dietary modification for control of diabetes?

(1) Yes  (2) No  (If No, go to question no. 45)

37. In a typical week, on how many days do you eat fruit?

38. How many servings of fruit do you eat on one of those days?

39. In a typical week, on how many days do you eat vegetables?

40. How many servings of vegetables do you eat on one of those days?

41. How much salt is consumed by your family per month?

42. What type of oil is usually used for meal preparation in your household?

(1) Saturated oil  (2) Unsaturated  (3) Mixed

43. How many liters of oil does your family consume in a month?

Physical activity assessment:

44. Does your work involve vigorous activity like lifting heavy loads, digging, and construction?

(1) Yes  (2) No (If No, go to question no. 48)

45. In a typical week, how many days do you do vigorous activities as a part of your work?

46. How much time do you spend on vigorous work on a typical day?

47. Does your work involve moderately intense activity that causes small increases in breathing or heart rate (such as brisk walking or carrying light loads) for at least 10 minutes?

(1) Yes  (2) No (If No, go to question no. 51)

48. In a typical week, how many days do you spend on moderate-intensity activities as a part of your work?

49. How much time do you spend doing moderate-intensity activities at work on a typical day?

50. Do you walk or use a bicycle for at least 10 minutes to go from place to place?

(1) Yes  (2) No (If No, go to question no. 54)

51. In a typical week, how many days do you walk or use a bicycle for at least 10 minutes continuously to travel?

52. How much time do you spend walking or bicycling for travel on a typical day?

53. Are you involved in any sports, fitness, or recreational (leisure) activities?

(1) Yes  (2) No (If No, go to question no. 57)

54. In a typical week, how many days do you do such sports, fitness, or recreational activities?

55. How much time do you spend doing such activities on a typical day?

56. Do you follow any measures for stress management?

(1) Yes  (2) No

57. If yes, which measure do you follow for stress management?

(1) Yoga  (2) Reading  (3) Gardening  (4) Painting  (5) Others (specify) ____

58. How frequently do you do the above activities in a week?

(1) Every day  (2) Every other day  (3) Twice or thrice a week  (4) Once a week

59. Physical measurement: waist circumference ____
